# Diversity of Dipteran Vectors Associated with Different Mammalian Hosts in a Peri-Urban Zoological Garden in NW Spain

**DOI:** 10.1007/s11686-026-01375-1

**Published:** 2026-08-24

**Authors:** Alejandro Polina, Yasmina Martínez-Barciela, Josefina Garrido

**Affiliations:** Departamento de Ecoloxía e Bioloxía Animal, Facultade de Bioloxía, Universidade de Vigo, Vigo, Spain

**Keywords:** *Culicoides*, Galicia, Mammal species, Mosquitoes, Sand flies, Zoological park

## Abstract

**Purpose:**

This study aims to characterise the different dipteran vector communities present at a peri-urban zoological and to explore differences in community composition among the different mammalian hosts, and to assess their potential epidemiological risk.

**Methods:**

Five animal enclosures were sampled and compared using CDC light traps equipped with white lights (WL) and ultraviolet traps (UV), used alternatively over five weeks in the summer of 2025.

**Results:**

A total of 598 dipteran vectors were collected in the present study: 495 mosquitoes belonging to nine species; 73 sand flies to one species; and 30 biting midges (*Culicoides* spp.) to six species. Generalized linear models (GLMs) showed a strong influence of larger mammals (sheep IRR = 7.28; bear IRR = 6.35) on mosquito abundance, registering more captures in comparison to meerkats (reference); as well as of the type of light, as UV captured up to 2.54 more specimens than WL. Sand fly abundance was mainly defined by the nearest host, as sheep (IRR = 49.52) and bear (IRR = 13.00) enclosures captured more specimens than the meerkat area. The non-metric multidimensional scaling ordination (nMDS) for the mosquito community showed that the animal enclosure explained 41% of the variance independently. Screening for bluetongue virus, epizootic haemorrhagic disease virus, and *Leishmania infantum* yielded negative results.

**Conclusions:**

The zoological garden of Vigo provides suitable conditions for the development of different vector species of mosquitoes, sand flies and biting midges, including invasive species such as *Ae. albopictus*. In addition, differences in vector composition were observed among host enclosures, providing valuable insights into vector ecology.

## Introduction

In recent years, an increasing number of autochthonous cases of emerging pathogens have been reported in Europe. Most of them correspond to vector-borne diseases (VBD), transmitted through the bite of infected arthropods. The rise in both the incidence and geographic distribution of these VBD is largely driven by two main factors. On the one hand, climate change is altering the distribution patterns of many vectors. On the other hand, globalization facilitates their spread and promotes invasions well beyond their native ranges [1, 2]. The order Diptera includes several of the most important vectors of major public health and veterinary health importance: (i) mosquitoes (Diptera: Culicidae), responsible for transmitting diseases such as malaria, dengue, and West Nile virus (WNV) to humans, as well as filarial nematodes and trypanosomes affecting several animals species [3–5]; (ii) *Culicoides* biting midges (Diptera: Ceratopogonidae), the main vectors of ruminant diseases such as bluetongue virus (BTV) and epizootic haemorrhagic disease (EHDV), and also vectors of trypanosomes and haemosporidian parasites infecting wildlife [6–8]; and (iii) sand flies (Diptera: Psychodidae), vectors of *Leishmania* spp. parasites and various phleboviruses affecting both humans and animals [9, 10].

Zoological gardens represent unique ecosystems where native and exotic fauna coexist in captivity within a variety of artificial microhabitats. In addition, human presence is constant, as staff and visitors frequent these areas daily. Zoos also provide suitable breeding sites for the abovementioned groups, since containers holding water, ponds, fresh manure, and muddy areas are commonly present. For these reasons, zoos constitute favourable environments for the development and survival of vectors [11, 12]. Their occurrence may negatively affect both animal and human welfare, causing irritation and discomfort, and potentially transmitting pathogens of concern [12].

Over the last few years, several studies have investigated the epidemiological role of zoological gardens and wildlife parks, as well as the ecology of hematophagous vectors in these environments [13–17]. Zoos can act as reliable early warning systems for the introduction and detection of emerging infectious diseases through the study of their vector communities and disease surveillance activities [13, 14]. In the Bronx Zoo (United States), Ludwig et al. assessed the seroprevalence of WNV in several bird species, finding that 34% of the tested animals were positive for WNV antibodies [15]. In the United Kingdom, England et al. characterised the *Culicoides* species assemblage and their host blood meals in two zoos, confirming the high activity and survival of this group and their ability to feed on a wide variety of native and exotic mammals [16]. In the Iberian Peninsula, Muñoz et al. described the composition and feeding patterns of sand flies in the Terra Natura wildlife park (Spain), underscoring the suitability of these environments for sustaining large populations of phlebotomines [17]. Additionally, Risueño et al. demonstrated the circulation of *Leishmania infantum* in wolves kept in captivity in a zoo [18].

The present study aims to characterise the communities of mosquitoes, sand flies, and biting midges in a peri-urban zoo in north-western Spain, focusing on their ecology and potential epidemiological risk through the analysis of pathogens of medical and veterinary relevance.

## Study Area

**Fig. 1:**
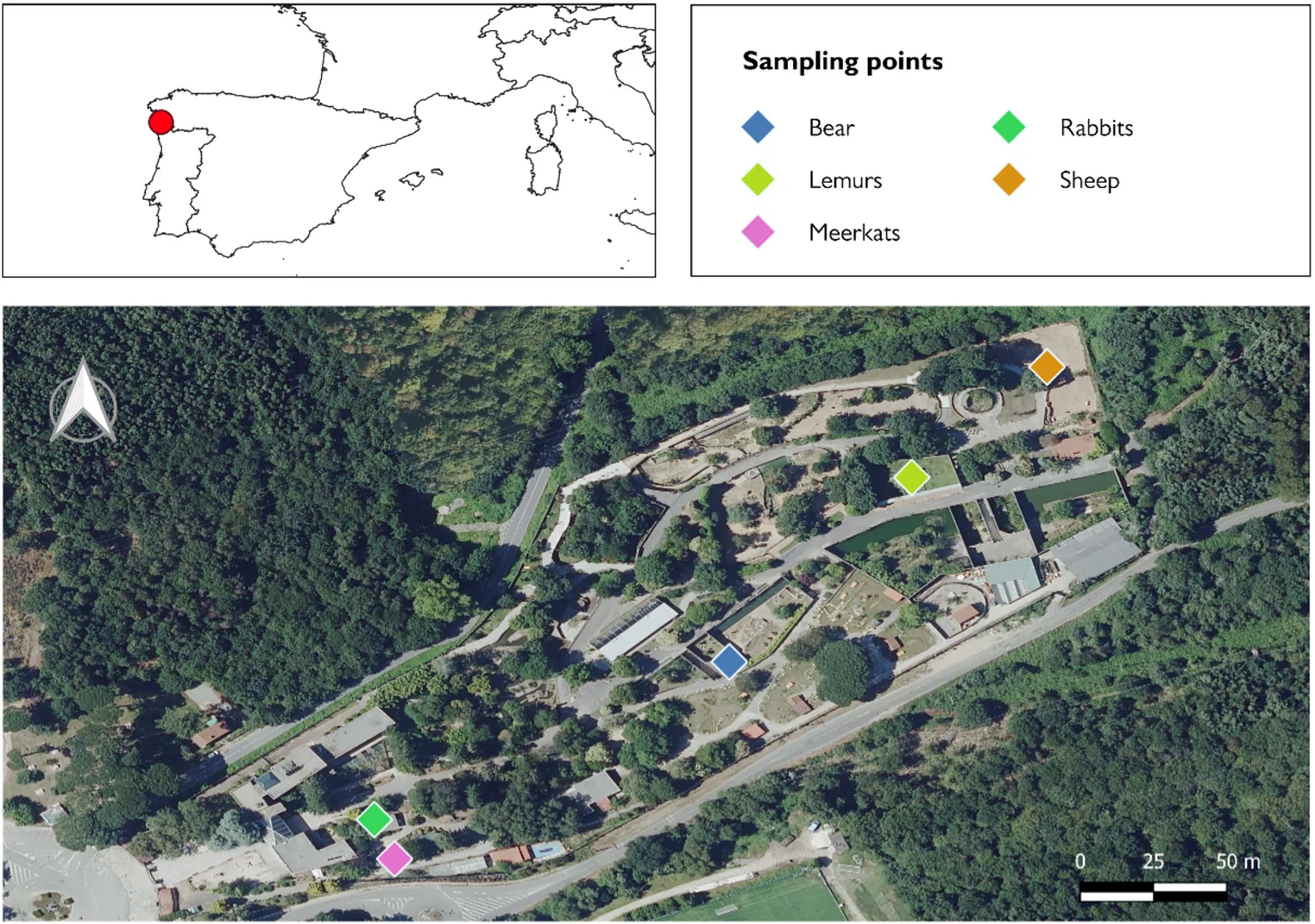
Top: location of the study area within the Iberian Peninsula. Bottom: satellite image of Vigo Zoo (Galicia), indicating the five sampled animal enclosures

The study was conducted at the Vigo Zoological Garden (Vigo Zoo), located in a peri-urban area at 319 m.a.s.l. in the surroundings of the city of Vigo, within the Autonomous Community of Galicia, the north-westernmost region of Spain (Fig. 1). The landscape is characterized by a mosaic of deciduous and coniferous trees, along with several artificial ponds distributed throughout the park. The surrounding area includes residential buildings and several sports facilities, as well as a dog shelter and a cemetery, both located at an approximately distance of 200 m. The urban centre lies about 3 km from the zoo, while the nearest urban settlement is situated approximately 500 m away. This region has a cool Mediterranean summer climate (Csb – oceanic temperate), according to the Köppen-Geiger Climate Classification (KCC). Galicia belongs to the Cantabrian coast, together with the Autonomous Communities of the Principality of Asturias, Cantabria, and the Basque Country. This area is commonly known as “Green Spain” because of its densely vegetated landscapes favoured by its oceanic climate.

Although the Vigo Zoo is currently transitioning to an environmental park, it still hosts approximately 500 animals belonging to 31 species in its outdoor area, covering an area of 6 ha. In addition to sporadic visitors and participants in outreach activities, the zoo receives daily visits from school groups. During the summer, it hosts several children’s camps and educational activities, attracting hundreds of visitors almost every day throughout this period.

### Sampling Procedure

**Fig. 2:**
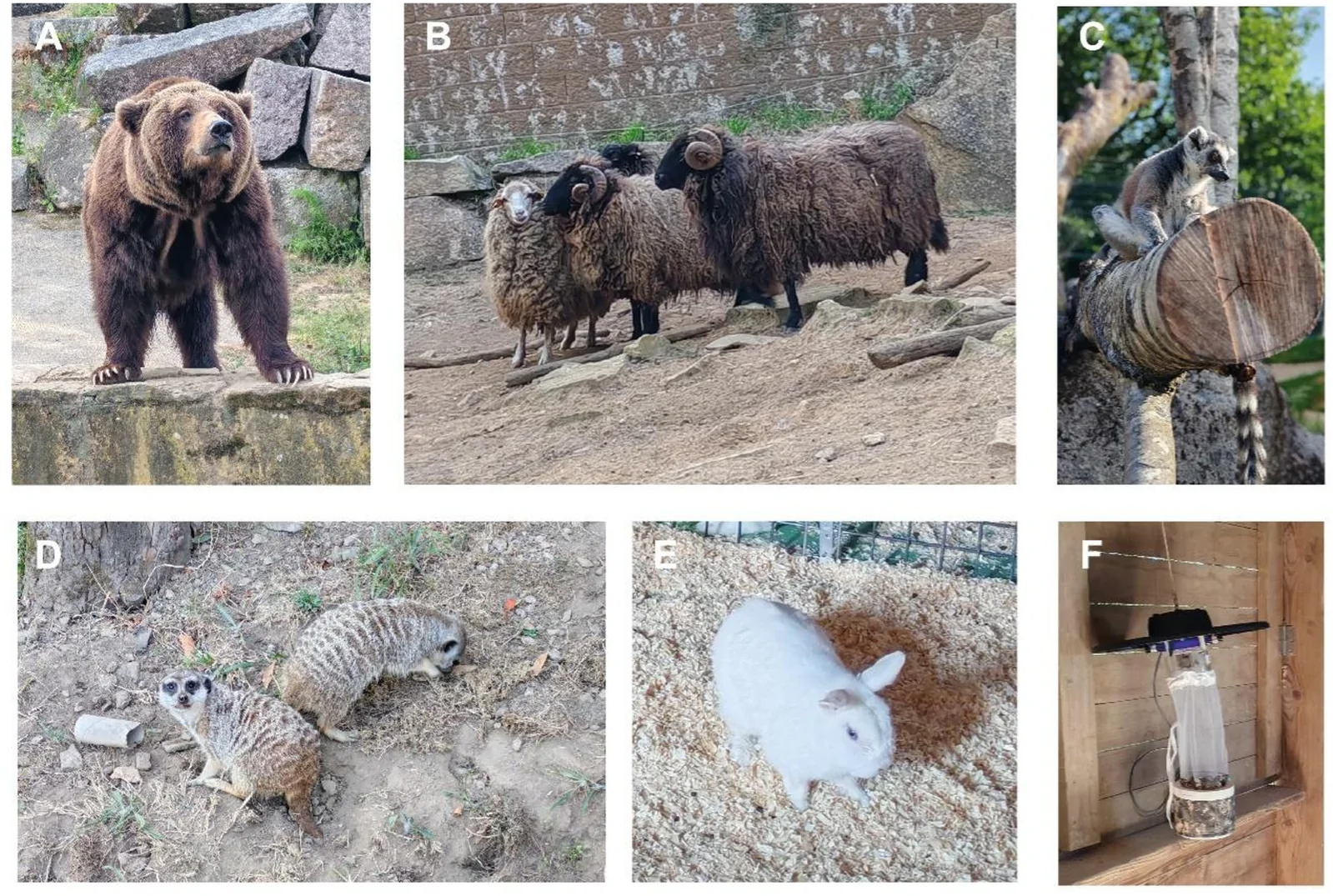
Mammal species present in the different enclosures sampled within the study area **A-E** and a CDC-UV light trap deployed in the rabbit area (**F**)

The sampling procedure was divided into three different trials corresponding to each month of early-mid summer of 2025 (June, July, and August). The first two trials consisted of consecutive daily collections over two weeks, excluding weekends due to park closure. The third trial consisted of only one week of daily sampling. CDC miniature light traps (John W. Hock Company) equipped with white lights (WL) or ultraviolet lights (UV) were deployed in five animal enclosures: bear, lemurs, meerkats, rabbits, and sheep (Fig. 2A-F). The criteria for site selection were related to host proximity, protection of the traps from sunlight and rainfall, and logistical reasons. Traps were hung at a height of 1.2–1.5 m, operated from dusk (17–18 h) to dawn (9–10 h), and maintained at distances ranging from a minimum of 15 m (meerkats-rabbits) to a maximum of 110 m (bear-lemurs). Traps were rotated daily among enclosures to ensure an equal number of replicates and to reduce potential methodological biases.

Samples were transported to the laboratory, where dipteran vector groups were separated from the rest of the insects and, when possible, morphologically identified to species level on a cold surface to prevent DNA denaturalization and potential loss of viral RNA. Specimens that could not be reliably distinguished based on morphological features were classified at complex level. The identification procedure varied according to the vector group. Both male and female mosquitoes were identified by their external morphology using identification keys provided by Becker et al. and Schäffner et al. [3, 19]. *Culicoides* biting midges were determined based on their wing patterns and features of the third segment of the maxillary palp, following identification keys provided by González & Goldarazena and Mathieu et al. [6, 20]. Male sand flies were identified through the examination of their genitalia, whereas females were determined by dissecting their three terminal abdominal segments, which were slide-mounted and cleared with Marc-André solution to visualise the spermathecae, following the identification keys of Gállego-Berenguer et al. and Prudhomme et al. [21, 22]. After identification, *Culicoides* females, sand flies females, and selected mosquito females were pooled by species, collection date, and enclosure, and stored at -80 °C until further analyses (Table 1). Number of specimens per pool ranged from one to four individuals. Engorged females were analysed individually. These samples were sent to Science & Business company (Vigo, Spain) for DNA/RNA extraction and pathogen detection.

**Table 1:** Number of pools and specimens analysed for pathogen detection, grouped by species

Species	Pools	Specimens	Pathogen
*Ae. geniculatus*	1	1	BTV & EHDV
*C. lupicaris*	2	2	
*C. newsteadi*	1	1	
*C. obsoletus* s.l.	5	11	
*C. pulicaris*	4	6	
*C. punctatus*	1	1	
*P. perniciosus*	12	16	*L. infantum*

## RNA/DNA Extraction

Viral RNA was extracted using the virus-specific kit Maxwell 16 Viral Total Nucleic Acid Purification Kit (Promega) in a Maxwell automatic extraction robot, following the manufacturer’s protocol [23]. Using a sterilised bistoury, the heads and thoraces of the mosquitoes were homogenised in the extraction tubes to ensure efficient lysis of the tissues, including the mouthparts and salivary gland region. Immediately, each pool was digested with proteinase K prior to viral extraction using the kit.

For the DNA extraction of *L. infantum*, sand fly individuals from each pool were homogenised using a sterilised mortar (Argos Technologies) and processed according to the protocol of Bruford et al. [24].

## Pathogens Detection Through RT-PCR

The Bio-T BTV&EHDV all genotypes kit (Biosellal) was used for bluetongue virus (BTV) and epizootic haemorrhagic disease virus (EHDV) detection. The kit allows the detection of both viruses within the same reaction, using specific oligonucleotides and fluorescently labelled probes that hybridise with conserved target regions. The kit allows reverse transcription from RNA to cDNA, followed by RT-qPCR amplification. Each virus has a unique fluorescent marker: FAM for BTV, VIC for EHDV, and an internal endogen control (gapdh) with Cy5 marker for the determination of enough cells and the absence of PCR inhibitors.

For *L. infantum*, the FLC2/RLC2 primer set was used in the RT-qPCR, as described by Gualda et al. [25].

### Data Analysis

Statistical analyses were performed using R statistical software version 4.4.2 [26].

## Diversity Indices Analysis

In addition to the abundance (N, total number of specimens) and species richness (S, total number of species) for each vector group, several diversity indices were calculated to quantify species diversity within the community using the BiodiversityR package [27]. Shannon-Wiener index (H^0^) measures species diversity by integrating both species richness and the proportional abundance of each species in the sample. Simpson’s dominance index (D) reflects the probability that two individuals randomly selected from the same community belong to the same species.

### Generalized Linear Models (GLM)

Associations between the abundance and species richness for each dipteran vector group with different qualitative variables were assessed through a generalized linear model (GLM). The explanatory variables included light source (WL and UV), host enclosure (bear, lemurs, meerkats, rabbits, and sheep), week of sampling (1 to 5), and month of sampling (June, July, and August). Given the strong overdispersion in the abundance data (variance greater than mean), negative binomial generalized linear models (NBGLM) were fitted using the *glm.nb* function from the MASS package [28]. A log-link function was applied, allowing the estimation of incidence rate ratios (IRRs) and their 95% confidence intervals (CI) for each category of the independent variables.

Model construction followed a backward elimination approach, starting from a saturated model that included all potential predictors. Non-significant variables were sequentially removed, and the final models retained only predictors showing statistical significance (*p* < 0.05).

## Non-Constrained Ordination Analysis

A non-metric multidimensional scaling (nMDS) ordination was performed to describe patterns in mosquito community composition across sampling sites. This analysis is particularly appropriate for ecological community matrices because it relies on rank-order similarities and is robust to non-linear species-environment relationships. The dataset comprised 100 sampling events and was divided into two matrices: (i) a community matrix including species abundance data and (ii) an environmental matrix containing potential qualitative explanatory variables (light source and host enclosure). Samples with no recorded individuals were removed from the dataset as they do not provide ecological information and may increase ordination stress.

Prior to analysis, the Hellinger transformation was applied to the community matrix to reduce the influence of the most abundant species. Given the large number of zeros in the dataset, resulting from species absences, the Bray-Curtis dissimilarity index was selected to perform the nMDS analysis. The nMDS was run in two dimensions to balance interpretability with ordination accuracy, and multiple random starts with up to 100 iterations were allowed to ensure convergence toward a stable solution. Ordination quality was assessed by inspecting the final stress value.

Subsequently, a post hoc vector fitting procedure (envfit) was applied to the resulting ordination to explore the association between community patterns and environmental variables. Only qualitative variables that were statistically significant (*p* < 0.05) after 999 permutations were retained for interpretation. All analyses were performed using the vegan package in R software [29].

## Results

A total of 598 dipteran vectors were collected across the five sampled enclosures (Table 2). Most specimens were mosquitoes (*N* = 495) belonging to nine species. *Culex pipiens* s.l. Linnaeus was the dominant species, representing 48.1% of the total culicid fauna (*N* = 238), followed by *Culex hortensis* Ficalbi (*N* = 113) and *Culiseta longiareolata* (Macquart) (*N* = 98), accounting for 22.8% and 19.8% of the total, respectively. Less abundant species included *Anopheles plumbeus* Stephens (*N* = 15; 3.0%), *An. maculipennis* s.l. Meigen (*N* = 9; 1.8%), *Aedes albopictus* (Skuse) (*N* = 5; 1.0%), *Culex theileri* Theobald (*N* = 4; 0.8%), *Culiseta annulata* (Schrank) (*N* = 4, 0.8%), and *Aedes geniculatus* (Olivier) (*N* = 1; 0.2%). Eight additional *Culex* specimens were too damaged to allow identification to species level.

**Table 2:** Abundance of dipteran vector species trapped in the five sampled animal enclosures. Unidentifiable specimens were not included. M = Males; F = Females; T = Total; % = Relative abundance

Mosquito species	Bear				Lemurs				Meerkats				Rabbits				Sheep				Total			
	M	F	T	%	M	F	T	%	M	F	T	%	M	F	T	%	M	F	T	%	M	F	T	%
*Ae. albopictus*	0	4	4	2,5	0	0	0	0,0	0	0	0	0,0	0	0	0	0,0	0	1	1	0,6	0	5	5	1,1
*Ae. geniculatus*	0	0	0	0,0	0	0	0	0,0	0	0	0	0,0	0	0	0	0,0	0	1	1	0,6	0	1	1	0,2
*An. maculipennis* s.l.	0	1	1	0,6	0	0	0	0,0	0	0	0	0,0	0	0	0	0,0	0	8	8	4,8	0	9	9	1,9
*An. plumbeus*	0	0	0	0,0	0	0	0	0,0	0	0	0	0,0	0	0	0	0,0	0	15	15	9,1	0	15	15	3,2
*Cx. hortensis*	2	4	6	3,8	18	13	31	57,4	13	4	17	73,9	21	33	54	76,1	3	2	5	3,0	57	56	113	23,9
*Cx. pipiens* s.l.	3	88	91	57,2	3	12	15	27,8	0	1	1	4,3	0	2	2	2,8	7	107	114	69,1	13	210	223	47,2
*Cx. theileri*	0	0	0	0,0	0	0	0	0,0	0	0	0	0,0	0	0	0	0,0	0	4	4	2,4	0	4	4	0,8
*Cs. annulata*	0	0	0	0,0	0	0	0	0,0	0	0	0	0,0	0	0	0	0,0	0	4	4	2,4	0	4	4	0,8
*Cs. longiareolata*	25	32	57	35,8	3	5	8	14,8	4	1	5	21,7	13	2	15	21,1	3	10	13	7,9	48	50	98	20,8
Total	30	129	159	100,0	24	30	54	100,0	17	6	23	100,0	34	37	71	100,0	13	152	165	100,0	118	354	472	100,0
***Culicoides*** ** species**																								
*C. festivipennis*	0	1	1	100,0	0	0	0	0,0	0	0	0	0,0	0	0	0	0,0	0	0	0	0,0	0	1	1	3,3
*C. lupicaris*	0	0	0	0,0	0	0	0	0,0	0	0	0	0,0	0	1	1	100,0	0	1	1	3,6	0	2	2	6,7
*C. newsteadi*	0	0	0	0,0	0	0	0	0,0	0	0	0	0,0	0	0	0	0,0	0	1	1	3,6	0	1	1	3,3
*C. obsoletus* s.l.	0	0	0	0,0	0	0	0	0,0	0	0	0	0,0	0	0	0	0,0	0	15	15	53,6	0	15	15	50,0
*C. pulicaris*	0	0	0	0,0	0	0	0	0,0	0	0	0	0,0	0	0	0	0,0	0	10	10	35,7	0	10	10	33,3
*C. punctatus*	0	0	0	0,0	0	0	0	0,0	0	0	0	0,0	0	0	0	0,0	0	1	1	3,6	0	1	1	3,3
Total	0	1	1	0,0	0	0	0	0,0	0	0	0	0,0	0	1	1	0,0	0	28	28	100,0	0	30	30	100,0
**Sand flies species**																								
*P. perniciosus*	10	3	13	100,0	4	2	6	100,0	1	0	1	100,0	1	0	1	100,0	40	12	52	100,0	56	17	73	100,0

Regarding *Culicoides* biting midges, 30 individuals from six species were captured, most of them in the sheep enclosure. Half of these specimens were *Culicoides obsoletus* s.l. (Meigen) (*N* = 15), followed by *Culicoides pulicaris* (Linnaeus) (*N* = 10; 33.3%). The remaining species were collected in very low numbers: *Culicoides lupicaris* Kettle & Lawson accounted for two specimens (6.7%) and *Culicoides festivipennis* Kieffer, *Culicoides newsteadi* Austen, and *Culicoides punctatus* (Meigen) accounted for one individual each.

A total of 73 sand flies were identified, all of them belonging to a single species: *Phlebotomus perniciosus* Newstead. Sand flies were found in every animal enclosure, although the sheep area recorded the highest number of individuals.

### Species Diversity and Richness

The nine mosquito species documented in this study were all present in the sheep enclosure. Lemur, meerkat, and rabbit enclosures showed the lowest species richness, with only three species collected. The sheep enclosure exhibited the highest diversity (H^0^ = 1.10), whereas the lowest value was observed in the rabbit area (Table 3). Regarding dominance, similar values were found in rabbit and meerkat enclosures (D = 0.62 and D = 0.60, respectively), indicating that a small number of species are dominating the community. In contrast, lemur and bear enclosures showed the lowest dominance values (D = 0.43 and D = 0.46, respectively).

**Table 3:** Total abundance (N), species richness (S), Shannon-Wiener index (H^0^), and Simpson’s dominance index (D) for mosquitoes (M) and *Culicoides* biting midges (C) in each host enclosure

Metrics	Bear		Lemurs		Meerkats		Rabbits		Sheep		Total	
	M	C	M	C	M	C	M	C	M	C	M	C
N	159	1	54	0	23	0	71	1	165	28	472	30
S	5	1	3	0	3	0	3	1	9	5	9	6
H^0^	0,94	0	0,96	0	0,69	0	0,64	0	1,1	1,06	1,33	1,23
D	0,46	1	0,43	0	0,6	0	0,62	1	0,53	0,42	0,33	0,37

† M = Mosquitoes; C = *Culicoides*

For biting midges, only three enclosures registered their presence: bear, rabbit, and sheep. The first two yielded only one individual each, whereas the highest numbers were collected in the latter. Consequently, five species were identified in the sheep area, resulting in the highest diversity (H^0^ = 1.06) (Table 3).

Regarding sand flies, as only a single species was found across the entire study area, no diversity indices could be performed for this group.

### Relationship Among Qualitative Variables and Vector Abundance/Richness

Multivariate models revealed statistically significant relationships between mosquito abundance, host enclosure, and light source. No significant associations were detected when sampling week or month were included as factors. Incidence rate ratios were significantly higher for all animal enclosures compared to meerkat’s, with the strongest associations observed for sheep (IRR = 7.28) and bear (IRR = 6.35). Regarding light source, UV traps collected 2.54 times more mosquitoes than WL traps (*p* < 0.05) (Table 4).

**Table 4:** Generalized linear model (GLM) results for the abundance and richness of mosquitoes in the study area. Predictors with their correspondent coefficients are shown: IRR = Incidence rate ratio; CI 95% = 95% confidence interval

Variables	Mosquito abundance		Mosquito richness	
	IRR (CI 95%)	*P*-value	IRR (CI 95%)	*P*-value
Intercept	0.67 (0.36–1.23)	0.20	0.44 (0.26–0.79)	0.01
Host enclosure				
Meerkats	Reference category		Reference category	
Bear	6.35 (3.20-12.84)	2.00e^07^	3.90 (2.02–8.26)	1.23e^− 04^
Lemurs	2.31 (1.12–4.81)	0.02	2.27 (1.11–4.97)	0.03
Rabbits	3.20 (1.58–6.60)	1.28e^− 03^	2.24 (1.10–4.91)	0.03
Sheep	7.28 (3.69–14.61)	1.54e^− 08^	5.30 (2.83–11.03)	1.20e^− 06^
Light source				
White	Reference category		Reference category	
Ultraviolet	2.54 (1.71–3.78)	3.67e^− 06^	1.31 (0.95–1.80)	0.09

The same factors were retained in the final model when analysing mosquito species richness. Positive associations were observed for all host enclosures when compared to the meerkat area. The greatest differences were recorded for sheep (IRR = 5.30) and bear (IRR = 3.90). Light source was also correlated with species richness, with UV traps capturing a higher species number than WL devices (IRR = 1.31), although this effect was only marginally significant (*p* < 0.10) (Table 4).

For sand fly abundance, the final model retained only the animal enclosure as a statistically significant variable. Higher values were observed for sheep (IRR = 49.52) and bear (IRR = 13.00) enclosures (*p* < 0.05) compared to the meerkat area. A positive association was also detected for the lemur enclosure (IRR = 5.71), although with lower statistical support (*p* < 0.20). No significant effect was observed regarding the rabbit enclosure (P-value = 0.99) (Table 5).

**Table 5:** Generalized linear model (GLM) results for the abundance of sand flies in the study area. Predictors with their correspondent coefficients are shown: IRR = Incidence rate ratio; CI 95% = 95% confidence interval

Variables	Sand flies abundance	
	IRR	*P*-value
Intercept	0.05 (2.75e^03^-0.24)	3.81e^− 03^
Host enclosure		
Meerkats	Reference category	
Bear	13.00 (2.14-252.77)	0.02
Lemurs	5.71 (0.83-115.27)	0.13
Rabbits	0.95 (0.04–25.68)	0.99
Sheep	49.52 (8.85-936.34)	2.91e^− 04^

Models could not be fitted for biting midge abundance or species richness, as nearly all individuals of this group were collected in the sheep enclosure. However, additional models restricted to the sheep area were performed, including light source, sampling week, and sampling month as potential explanatory variables. None of these variables were retained in the final models at a significance level of *p* < 0.20.

### Non-Constrained Ordination Analysis for Mosquito Community

**Fig. 3:**
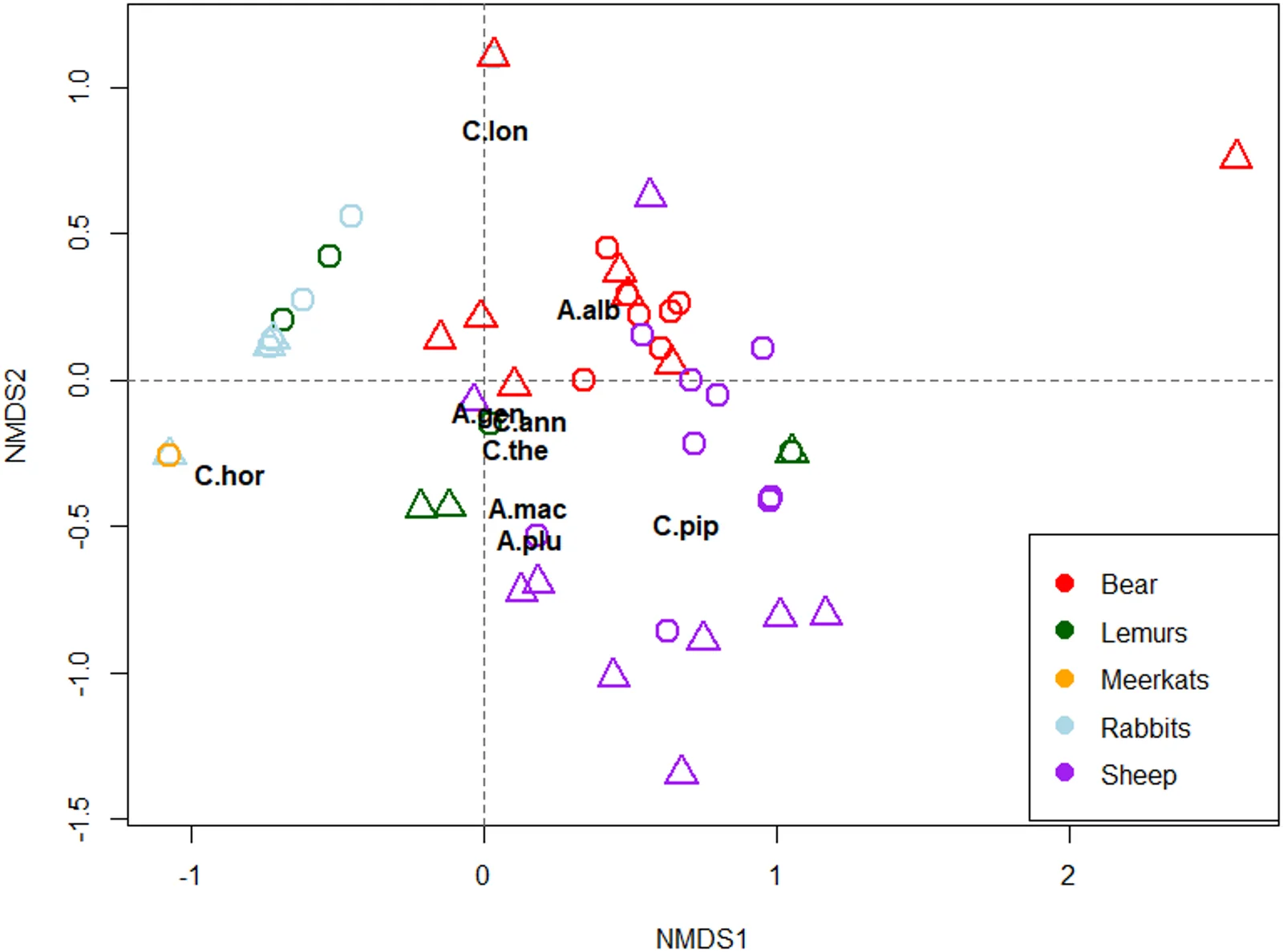
Graphical representation of the non-constrained ordination analysis nMDS for the mosquito community composition across sampling sites. Triangles: white light traps; Circles: ultraviolet light traps

The nMDS analysis produced a two-axis optimal solution (Fig. 3) with a final stress value of 0.078, indicating a high-quality model fit. The environmental variables included in the assessment were host enclosure and light source. The host enclosure variable showed an adequate fit and alone explained 41% of the variance. In contrast, light source had no detectable effect on the mosquito community, accounting for less than 1% of the variance and failing to reach statistical significance (Table 6).

**Table 6:** Centroid values for each variable included in the nMDS analysis, together with their goodness of fit. r^2^ = proportion of variation explained by each variable

	nMDS1	nMDS2
Host enclosure		
Bear	0.521	0.321
Lemurs	−0.175	−0.047
Meerkats	−0.636	0.038
Rabbits	−0.714	0.092
Sheep	0.627	−0.351
Light source		
Ultraviolet	0.005	0.040
White	−0.005	−0.041
**Goodness of fit**	**r** ^**2**^	**P-value**
Host enclosure	0.413	0.001
Light source	0.002	0.840

The close proximity of the centroids for the meerkat and rabbit enclosures suggests similar community structure between both groups. In contrast, the centroids for the bear and sheep enclosures are widely separated and oriented in opposite directions, indicating dissimilar species composition. The centroid for the lemur enclosure is located near the origin, reflecting an intermediate community structure in comparison to the other groups. Regarding light source, the centroids clustered tightly, thereby preventing the detection of meaningful differences between groups (Fig. 3; Table 6).

The ordination plot shows a strong relationship between *Cx. pipiens* s.l. and the sheep enclosure. *Anopheles maculipennis* s.l. and *An. plumbeus* also cluster near this animal area as well, forming a distinct assemblage. *Aedes geniculatus*, *Cs. annulata*, and *Cx. theileri* are positioned near the origin, showing only a slight tendency towards the sheep assemblage, although no clear associations could be inferred. Conversely, *Ae. albopictus* shows close proximity to the bear enclosure, resulting in a marked association. The plot also suggests a strong relationship between *Cx. hortensis* abundance and the meerkat and rabbit enclosures. *Culiseta longiareolata* is primarily associated with the NMDS2 axis, lying closer to the bear centroid (Fig. 3; Table 2).

### Pathogens Detection

Fourteen pools of biting midges and mosquitoes were tested for BTV and EHDV, and 12 pools of sand flies were screened for *L. infantum*. All 26 pools resulted negative.

## Discussion

The present study allowed the characterisation of the dipteran vector communities in a zoological garden in northwestern Spain, together with an assessment of their potential role in pathogen transmission. To date, this represents the first investigation conducted in such a particular ecosystem within the Cantabrian coast and one of the most comprehensive studies on this topic carried out in the Iberian Peninsula.

### Mosquitoes

Nine mosquito species were identified in the present study. Direct comparisons with other investigations on vector density and ecology in zoological gardens or wildlife parks are difficult, as sampling designs vary considerably among them. For instance, Martínez-de la Puente et al. reported three mosquito species in an urban zoo in Barcelona (Spain), where BG-Sentinel 2 traps and entomological aspirators were used [30]. Madeira et al. conducted a survey in an urban zoo in Lisbon (Portugal) using CDC-light traps as the sampling method and recorded five species, albeit at relatively low densities [12]. Conversely, Heym et al. surveyed two zoos in Germany within the same climatic region using EVS-light traps and collected 16 different taxa, with a higher diversity observed in the rural zoo [11]. Several studies have reported positive gradients in mosquito abundance and diversity from urban to rural environments, which is consistent with the findings of the present study [31, 32]. In addition, CDC-light traps have proven effective in reflecting this vector diversity, as previously reported by other authors [33].

Generalized linear models revealed several statistically significant differences among host enclosures. All animal enclosures showed higher mosquito abundance and species richness compared to the meerkat area, although those differences were uneven. Whereas enclosures hosting smaller animals, such as lemurs and rabbits, registered 2.3 and 3.2 times more specimens, respectively, substantially greater abundances were observed in enclosures hosting larger mammals, reaching 6.3 times and 7.3 more individuals in the bear and sheep area, respectively. A similar pattern was observed for species richness, as IRRs increased to 3.9 and 5.3 for the bear and sheep areas, respectively, when compared to meerkats. Blood-meal analyses in previous studies have shown that DNA from large mammals is detected more frequently in the blood meals of several mammophilic mosquito species [34, 35]. In addition, mammophilic mosquitoes are known to respond to host-associated factors including body temperature, carbon dioxide (CO_2_) emitted during respiration, and carboxylic acid released in mammalian sweat [3]. Therefore, the greater abundance observed in enclosures housing larger mammals may reflect the higher attractiveness of these hosts for mammal-feeding mosquito species. Nevertheless, host-vector interactions are highly specific, and these findings should be interpreted with caution.

Regarding light sources, models also confirmed that UV outperformed WL in capturing higher mosquito densities. Specifically, UV traps collected up to 2.5 times as many mosquitoes as WL traps. Although weaker, a similar pattern was observed for species richness, as UV collected up to 1.3 times more species than WL. These findings are consistent with observations made by other authors [36, 37]. Nevertheless, other comparative studies have reported no significant differences between UV and WL traps, suggesting that the relative effectiveness of light sources may depend on environmental conditions, mosquito community composition, or trap design [38, 39].

In terms of mosquito species composition, the dominance of the Pipiens complex aligns with previous findings identifying it as the most widespread culicid in Galicia [40, 41], particularly in areas associated with farmland animals [42]. Similar patterns were observed in other zoological gardens within the Iberian Peninsula, suggesting the presence of mammophilic biotypes [12, 30]. Although the species was collected in all sampled enclosures, its abundance varied considerably. Small mammals (lemurs, meerkats, and rabbits) did not appear to favour its presence, whereas enclosures housing larger mammals (bear and sheep) recorded the highest numbers. This pattern is consistent with the non-constrained ordination analysis, which revealed a strong correlation between the sheep enclosure and species abundance. The species affinity for ovine hosts has already been documented by Bravo-Barriga et al. [43]. Additionally, numerous artificial ponds and containers within the study area provide optimal breeding habitats for the species [40]. Together, these factors likely explain the marked dominance of the species within the zoological garden.

A noteworthy result was the high abundance of *Cx. hortensis*, which emerged as the second most common mosquito species in the study area. Although the Mediterranean basin lies within its distribution range [3], its frequency in the Iberian Peninsula seems to follow a latitudinal pattern, as it is a more common species in northern regions than in southern ones [44]. The species was found across all enclosures, but unlike *Cx. pipiens* s.l., it was more numerous in areas with small mammals, a pattern supported by the nMDS analysis, which associated it with the meerkat and rabbit enclosures. A host preference of *Cx. hortensis* for small mammals has not been documented, as the species is generally considered to feed primarily on reptiles and amphibians [19]. However, recent studies have suggested an opportunistic feeding behaviour, with the ability to feed on a wide range of vertebrates, including small mammals such as cats [34]. Moreover, its high adaptability to breed in both natural and artificial aquatic ecosystems [40, 45] may further favour its development and proliferation in the study area.

*Culiseta longiareolata* was the third most abundant species recorded and occurred in all five enclosures, with the highest numbers observed in the bear area. Although the non-constrained ordination analysis suggested a slight shift toward this enclosure, the association was not clear. The species has been documented to feed on mammals, including humans and larger mammals [46, 47].

The invasive species *Ae. albopictus* was detected in two locations, in the bear and sheep enclosures. First recorded in the region in 2023 [48], the species has rapidly colonized several Galician municipalities and a wide variety of habitats [41]. *Aedes albopictus* is capable of feeding on a broad range of vertebrates [49, 50], and the zoo likely provides favourable conditions for its proliferation considering the regular presence of both human and animal hosts. The nMDS analysis showed a marked association between the species and the bear enclosure, suggesting a potential attraction to this host. Moreover, the inefficiency of CDC light traps in capturing *Ae. albopictus* [51, 52] may be underestimating the true abundance of its populations, highlighting the need for surveillance using BG-Sentinel 2 traps.

All captures of the two *Anopheles* species recorded in this study, *An. plumbeus* and *An. maculipennis* s.l., were obtained in the sheep enclosure, with the exception of a single specimen of the Maculipennis complex collected in the bear area. Consequently, the clear association between ovine hosts and both anopheline species observed in the nMDS analysis is not unexpected. Both species are known to be attracted to mammals, including sheep and other livestock [53]. Furthermore, the peri-urban location of the zoo falls within the ecological preferences of these mosquitoes, which are particularly favoured in suburban and rural environments over urban ones [54–56].

*Culex theileri*, *Cs. annulata*, and *Ae. geniculatus* were recorded in very low numbers exclusively in the sheep enclosure, which is consistent with the results of the constrained ordination analysis. *Culex theileri* and *Cs. annulata* are known to feed on a wide range of vertebrates, including ovine hosts [47, 57]. Regarding *Ae. geniculatus*, its anthropophilic behaviour is well documented [58], and it is also known to be capable of feed on other vertebrate hosts such as cows or rabbits [59, 60], so the possibility to be attracted also by sheep cannot be discarded.

### Sand Flies

All 73 specimens of sand flies collected were identified as *P. perniciosus*, considered the main vector of *L. infantum* in the Iberian Peninsula [61]. Therefore, the GLM results for overall abundance may be interpreted as reflecting the behaviour of this species. In the present study, the presence of *P. perniciosus* was confirmed in every host enclosure, although significant differences were observed among them. While meerkats and rabbits are also potential hosts—and even potential reservoirs of *L. infantum—* [62–64], the models indicated higher captures in the lemur enclosure (significant at *p* < 0.15) and, particularly, in areas housing larger mammals, with sand fly abundance reaching values up to 13.0 and even 49.5 times higher in the bear and sheep enclosures, respectively, compared to meerkat enclosure. The presence of sand flies in the lemur enclosure suggests a potential attraction to them as blood sources, a hypothesis reinforced by recent evidence identifying lemurs as potential reservoirs of *L. infantum* [14, 65]. However, the strongest attraction appears to be exerted by the largest mammals. Sand flies are considered opportunistic feeders capable of biting a wide range of vertebrate species, typically favouring those with easier accessibility [66, 67], which may explain why bear and sheep could act as particularly suitable hosts for blood-feeding. Although no studies have directly documented sand fly blood meals from bears, previous research has shown their ability to feed on uncommon hosts such as tigers and rhinoceroses [68, 69]. Further evidence exists for ovine cattle, as several studies have reported a high affinity of *P. perniciosus* for small ruminants [70–72]. Moreover, a seroepidemiological survey of livestock in an endemic *L. infantum* focus in Southern Spain found that 10.8% of clinically healthy animals tested positive for antibodies, with sheep (15.6%) showing one of the highest seroprevalence values, suggesting that these animals may act as asymptomatic reservoirs and contribute to pathogen circulation [73]. Nevertheless, there is no transmission evidence in the study area, according to the pathogen screening carried out in sand flies.

Although WL traps collected 35.5% more individuals than UV traps (42 over 31 specimens, respectively), light source did not appear to influence sand fly capture abundance according to the GLM results even at a significance level of *p* < 0.20. Several authors have compared the performance of different light sources, with some reporting higher capture rates for UV over WL [74, 75]. The present study contrasts with those findings and, in particular, with observations by Sierpe-Jeraldo et al., who recommended the use of UV traps in areas with low sand fly densities [76]. However, other contextual factors, such as host proximity, should be considered as influencing sand fly capture rates. Consequently, the suitability of local environmental conditions should be carefully evaluated before issuing operational recommendations for sand fly surveillance.

### Biting Midges

Abundances of biting midges collected in the study area were much lower than expected, particularly when compared to other studies focusing on *Culicoides* collections in areas with regular presence of animals [16, 36]. Even in Galicia, previous research has reported a high prevalence of this vector group on livestock farms, especially species within the Obsoletus complex [77, 78]. Although the selected sampling dates fell within the optimal activity period for most dominant *Culicoides* species in the region (June to August) [71], the exceptional climatic conditions during the summer of 2025 in the study area may have reduced their presence. This season was atypically dry, with higher-than-average temperatures (on some days exceeding 33 °C) and extremely scarce precipitation [79]. Barceló & Miranda observed that *C. obsoletus* s.s. immature stages do not survive when temperatures exceed 30 °C [80]. Most *Culicoides* species require breeding habitats with sufficient moisture, such as fresh manure, mud, or decaying leaf litter [81]. In the absence of rainfall, such microhabitats were likely limited, reducing oviposition, larval development, and ultimately the emergence of adults. Therefore, the evidence obtained in the present study should be interpreted with caution and considered preliminary. Additional surveys conducted under more favourable climatic conditions would be necessary to better characterize the *Culicoides* community in the zoological park, as the site may support higher abundances and species diversity than observed during the analysed time period.

With this context in mind, several studies conducted in Europe analysing the host-feeding patterns of *Culicoides* species have identified ruminants as the most common hosts in blood meals [82–84]. Therefore, it is not surprising that the highest abundance was recorded in the sheep enclosure within the study area. When comparing the performance of both light trap sources in the sheep enclosure, no statistically significant differences were found in biting midges capture rates. However, the scarce number of captures may have prevented a reliable assessment of trap efficiency. Overall, the WL proved effective only for the collection of the Obsoletus complex and *C. pulicaris*. In contrast, UV additionally attracted *C. festivipennis*, *C. lupicaris*, *C. newsteadi*, and *C. punctatus*. The superiority of UV light in capturing broader diversity of *Culicoides* species has been consistently reported worldwide [36, 85, 86]. In particular, rare or infrequently collected species such as *C. festivipennis* or *C. newsteadi* are often detected exclusively using UV traps, as noted in previous studies [36, 38].

Finally, although the number of specimens collected was low, most of the recorded species are recognised or suspected vectors of BTV (*C. lupicaris*,* C. newsteadi*,* C. obsoletus* s.l., *C. pulicaris*,* C. punctatus*). For this reason, and considering the recent outbreaks of BTV and EHDV reported in the region [87, 88], collected specimens were screened for the presence of both viruses, but no evidence of pathogen circulation was detected within the study area.

## Conclusions

The present study represents a pioneering effort within an Atlantic-Mediterranean context as the first survey in the Iberian Peninsula to characterise the three main groups of dipteran disease vectors of diseases in a zoological setting, focusing on both public health and animal welfare. The zoological garden of Vigo provides suitable conditions for the development of different vector species of mosquitoes, sand flies and biting midges, including invasive species such as *Ae. albopictus*. In addition, differences in vector composition were observed among host enclosures, providing valuable insights into vector ecology. Although no pathogens were detected in the samples analysed, the presence of competent vectors highlights the need for continued surveillance, as these species may pose a risk and represent a nuisance for visitors, staff, and animals. 

## Data Availability

The data that support the findings of this study are openly available in Zenodo at https://doi.org/10.5281/zenodo.19252624.
